# Neuromuscular Adaptations to Multimodal Injury Prevention Programs in Youth Sports: A Systematic Review with Meta-Analysis of Randomized Controlled Trials

**DOI:** 10.3389/fphys.2017.00791

**Published:** 2017-10-12

**Authors:** Oliver Faude, Roland Rössler, Erich J. Petushek, Ralf Roth, Lukas Zahner, Lars Donath

**Affiliations:** ^1^Department of Sport, Exercise and Health, University of Basel, Basel, Switzerland; ^2^Department of Public and Occupational Health & Amsterdam Movement Sciences, VU University Medical Center, Amsterdam, Netherlands; ^3^College of Human Medicine, Michigan State University, East Lansing, MI, United States; ^4^Institute of Training and Computer Science in Sport, German Sport University Cologne, Köln, Germany

**Keywords:** exercise training, sensorimotor, leg strength, balance, power, efficacy, risk factor, team sport

## Abstract

**Objective:** Neuromuscular injury prevention programs (IPP) can reduce injury rate by about 40% in youth sport. Multimodal IPP include, for instance, balance, strength, power, and agility exercises. Our systematic review and meta-analysis aimed to evaluate the effects of multimodal IPP on neuromuscular performance in youth sports.

**Methods:** We conducted a systematic literature search including selected search terms related to youth sports, injury prevention, and neuromuscular performance. Inclusion criteria were: (i) the study was a (cluster-)randomized controlled trial (RCT), and (ii) investigated healthy participants, up to 20 years of age and involved in organized sport, (iii) an intervention arm performing a multimodal IPP was compared to a control arm following a common training regime, and (iv) neuromuscular performance parameters (e.g., balance, power, strength, sprint) were assessed. Furthermore, we evaluated IPP effects on sport-specific skills.

**Results:** Fourteen RCTs (comprising 704 participants) were analyzed. Eight studies included only males, and five only females. Seventy-one percent of all studies investigated soccer players with basketball, field hockey, futsal, Gaelic football, and hurling being the remaining sports. The average age of the participants ranged from 10 years up to 19 years and the level of play from recreational to professional. Intervention durations ranged from 4 weeks to 4.5 months with a total of 12 to 57 training sessions. We observed a small overall effect in favor of IPP for balance/stability (Hedges' g = 0.37; 95%CI 0.17, 0.58), leg power (g = 0.22; 95%CI 0.07, 0.38), and isokinetic hamstring and quadriceps strength as well as hamstrings-to-quadriceps ratio (g = 0.38; 95%CI 0.21, 0.55). We found a large overall effect for sprint abilities (g = 0.80; 95%CI 0.50, 1.09) and sport-specific skills (g = 0.83; 95%CI 0.34, 1.32). Subgroup analyses revealed larger effects in high-level (g = 0.34–1.18) compared to low-level athletes (g = 0.22–0.75), in boys (g = 0.27–1.02) compared to girls (g = 0.09–0.38), in older (g = 0.32–1.16) compared to younger athletes (g = 0.18–0.51), and in studies with high (g = 0.35–1.16) compared to low (g = 0.12–0.38) overall number of training sessions.

**Conclusion:** Multimodal IPP beneficially affect neuromuscular performance. These improvements may substantiate the preventative efficacy of IPP and may support the wide-spread implementation and dissemination of IPP. The study has been a priori registered in PROSPERO (CRD42016053407).

## Introduction

Physical inactivity is a major public health burden and an independent risk factor for non-communicable diseases as well as increased mortality (Blair, 2009; Kohl et al., 2012). Already in childhood, inappropriate physical activity levels can cause considerable health problems on individual as well as society level (Janssen and Leblanc, 2010; Andersen et al., 2011). Consequently, the World Health Organization recommends at least 60 min of moderate to vigorous physical activity on top of everyday physical activity to counter harmful cardiovascular, neuromuscular, and metabolic developments (WHO, 2010). As a side-effect, however, sport and high levels of physical activity are associated with a high prevalence of injuries (Caine et al., 2006; Emery, 2010). For instance, there is evidence from several countries (Switzerland, United States, Canada, France, the Netherlands, the United Kingdom and Sweden) that (organized and non-organized) sports is the main cause of injury in children and adolescents with more than 50% of all injuries caused by sports (Bijur et al., 1995; Mummery et al., 1998; Belechri et al., 2001; Michaud et al., 2001; Hedstrom et al., 2012).

In order to reduce injury incidence while being physically active and, therefore, supporting the beneficial health effects of sport and physical activity, injury prevention programs (IPP) have been developed and evaluated. There is convincing evidence that exercise-based prevention programs can reduce the overall injury rate by about 40% in child and adolescent sport (Rössler et al., 2014). IPP are usually designed as multimodal exercise interventions targeting potential deficits in neuromuscular abilities, such as, leg muscle strength and power or postural stability. Neuromuscular performance can be regarded as the ability of the neuromuscular system to functionally control and drive movements by an appropriate use and coordination of muscular strength and endurance, muscle recruitment pattern, proprioceptive feedback, and reflex activity (Huston and Wojtys, 1996; Zech et al., 2010). Neuromuscular deficits may potentially increase the risk of injury, although evidence in this regard is not conclusive to date (Bahr and Holme, 2003; Emery, 2003; Meeuwisse et al., 2007; Lehr et al., 2017). Successful prevention programs usually include exercises targeting static and dynamic balance, plyometrics, as well as lower limb strength and power (Mandelbaum et al., 2005; Abernethy and Bleakley, 2007; Soligard et al., 2008; Kiani et al., 2010). There are studies, which analyzed the extent to which potential neuromuscular risk factors for injuries were affected by such programs. Some of these studies, however, suffer from low sample sizes, the results appear heterogeneous and definitive conclusions on the expectable effect sizes of potential adaptations are not possible. A systematic analyses of the existing scientific literature in this regard is missing to date.

From a practical perspective, improvements of neuromuscular performance are relevant regarding sport-specific performance (Lesinski et al., 2015; Granacher et al., 2016). In consequence, performance improvements can be a relevant argument—next to the injury prevention perspective—to convince coaches and athletes to implement IPP in their training routine. As deficits in neuromuscular control are considered relevant risk factors for injuries to the lower limbs (Bahr and Holme, 2003; Meeuwisse et al., 2007; Alentorn-Geli et al., 2009; Frisch et al., 2009; Myer et al., 2011), data on adaptations in neuromuscular performance may additionally provide insights in mechanisms underlying the preventative efficacy of multimodal IPP. It can be further argued that the adaptive potential of athletes is limited at higher performance levels, as physical capacities are already well-developed. Therefore, it is of particular interest whether adaptations to prevention programs designed for a large mass of mainly recreational-level children and adolescents can be transferred to high-level youth athletes. Moreover, there is consistent evidence that age and sex affect injury risk (Emery, 2003; Frisch et al., 2009; Faude et al., 2013) and that training dose determines the size of training adaptations to neuromuscular training programs (Lesinski et al., 2015, 2016).

The aims of our systematic review with meta-analysis were: (a) To summarize the scientific literature on neuromuscular performance adaptations resulting from multimodal IPP in organized child and adolescent sport, (b) to quantify the effect sizes of adaptations in neuromuscular performance measures, and (c) to perform sub-group analyses in order to evaluate potential influences of performance level, sex, age-group, training volume, and potential differences between specific IPP. We hypothesized that multimodal, neuromuscular IPP can improve several neuromuscular performance parameters and that these effects were greater in low-level as compared to high-level youth athletes.

## Methods

We conducted and reported this systematic review in accordance with the Preferred Reporting Items for Systematic Reviews and Meta-Analyses (PRISMA) statement (Moher et al., 2009). The study has been a priori registered in PROSPERO (CRD42016053407). In contrast to the primary registration, we extended the age-range of includable youth athletes from 18 to a maximum of 20 years of age, as we noticed during the literature search process that a part of the athletes, particularly at the highest performance level, competing in the oldest youth age groups are older than 18 years and we aimed to present a comprehensive overview in organized youth sports.

### Literature search

A systematic literature search was conducted independently by two researchers (OF, LD) until May 8th, 2017 in the following electronic databases: PubMed, Web of Science, EMBASE, CINAHL, and SCOPUS. The search strategy was adopted using the PICOS (population, intervention, comparison, outcome, study design) approach. Selected search terms related to youth sports, injury prevention, and neuromuscular performance were combined in Boolean logic. No restriction with regard to publication date was applied. Only full-text articles written in English language were considered. Our search strategy is described in detail in Appendix 1 (Supplementary Material). In addition, we screened the reference lists of the selected articles as well as the authors' own bibliographies. Finally, we searched Google Scholar with the same search terms in order to control for potentially overseen relevant articles.

### Eligibility criteria and study selection

Inclusion criteria (according to the PICOS approach) were: (i) healthy participants, up to 20 years of age, who were involved in organized sport (club, high school, college, sports associations) (P), (ii) the study included an intervention arm performing a multimodal neuromuscular training program focusing on injury prevention (I) as well as a control arm following a common training or a sham treatment without a specific neuromuscular focus (C), (iii) at least one neuromuscular performance parameter (static or dynamic balance/stability, power, strength, sprint ability) was assessed (O), and (i) the study was designed as a (cluster-) RCT (S).

Exclusion criteria were: (i) any intervention outside organized sport (for instance, unorganized recreational or leisure time sport, physical education classes), (ii) any neuromuscular intervention without a specific injury prevention focus (e.g., general strength and conditioning programs focusing on performance enhancement), (iii) the control group performing another structured neuromuscular training program outside common training routine, and (iv) athletes suffering from systemic neurological or neuromuscular disease or disability or being injured or undergoing rehabilitation after injury. Studies were independently selected by two investigators (OF, LD). A final decision on eligibility was achieved by consensus.

### Data extraction and outcome parameters

Data extraction was independently performed by two investigators (OF, RoR). In case of discrepancies a third researcher (LD) was consulted. Relevant study information regarding author, year, number of participants, intervention (weeks, frequency, duration per session) and passive control condition were extracted and transferred to an excel spread sheet.

Main outcome parameters for this analysis were measures of neuromuscular performance, which have been associated with injury risk and/or which have been assumed to be associated with sport-specific performance. We analyzed parameters indicating balance/stability (sub-categories static and dynamic balance as well as dynamic stability, i.e., the ability to stabilize the center-of-pressure after a dynamic movement, e.g., a jump), leg power (basic and reactive vertical as well as horizontal jump performance), and strength (isokinetic hamstrings and quadriceps strength as well as strength ratios) as these measures might be related to injury risk (Lehr et al., 2017). In addition, we analyzed parameters related to sprint ability (basic straight sprint performance, acceleration, and change-in-direction speed) and sport-specific skills (here: soccer-specific skills like slalom dribbling and the wall-volley test). All analyzed parameters, the particular sub-categories as well as the specific tests, which were integrated in each sub-category, are presented in Appendix 2 (Supplementary Material). We purposely did not analyze potential anatomical or biomechanical risk factors for injuries, which are not directly related to sports performance (e.g., lower limb joint angles or moments or ground reaction forces). If more than one potential parameter for a particular sub-category was reported in a single study, we chose the following procedure for statistical analyses: (i) in case of two parameters (e.g., single leg stance with opened and closed eyes as an indicator of static balance performance) we used the one showing the smaller effect in order to arrive at a conservative estimate; (ii) in case of three parameters (e.g., if only the three reaching directions, but no average or composite score was reported in the Y-balance test) we used the reaching direction showing the medium effect for further analysis.

Means and standard deviations (SD) of pre- and post-tests were available in most studies and par for par extracted. In two studies (Vescovi and VanHeest, 2010; Reis et al., 2013), data were only available as graphs. In these two studies, means and SD were independently extracted from the figures by two investigators (OF, RoR) and the average value was used for further analysis. In three studies (Steffen et al., 2008, 2013; Zech et al., 2014), only pre-test values and change scores were available. In these cases, we calculated the post-test mean by adding the change score to the pre-test mean and used the pre-test SD for statistical analysis.

### Risk of bias assessment

The methodological quality of the included RCTs was rated using the PEDro scale. This scale comprises 11 dichotomous items (either yes or no). Studies were rated by two reviewers independently (LD and OF). After completing the evaluation, both examiners came to a consensus on every item. The raters were not blinded to study authors, place of publication, and results.

In order to examine a potential publication bias, we performed a risk-of-bias related sensitivity analysis between “weak” (score 5 and 6 on PEDro scale) and “strong” (score 7 on PEDro scale) studies for all main outcome parameters. Furthermore, we conducted a qualitative funnel plot evaluation.

### Meta-analysis

We conducted a quantitative synthesis of the included studies. We calculated the sample size adjusted standardized mean differences [Hedges' g with 95% confidence intervals (CI)] from pre- to post-test for each variable and for each study arm. The difference of the target outcome between the intervention and the respective control group including the pooled standard deviations was computed. Negative effects in favor of the control arm were symbolized with a minus sign. As we analyzed studies, which were basically different in many ways (e.g., regarding participants, interventions, researchers, etc.), data were analyzed using an inverse-variance model with random effects (Borenstein et al., 2010; Deeks and Higgins, 2010). We used the Cochrane Review Manager Software (RevMan 5.3, Cochrane Collaboration, Oxford, UK) for statistical analyses. Forest plots with 95% CI were created. The magnitude of g was classified according to the following scale: 0–0.19 = negligible effect, 0.20–0.49 = small effect, 0.50–0.79 = moderate effect and ≥0.80 = large effect (Cohen, 1988).

### Subgroup and exploratory analyses

We conducted a subgroup analysis with regard to different performance levels of participants. Thereby, performance level was classified as high, if it was explicitly stated that players competed on the highest youth level in the corresponding age group or on national level and that athletes trained three or more times per week with additional competitions at the weekend. Performance level was classified as low, if most players competed on a sub-elite level or that they trained maximally two times per week. Further exploratory analyses referred to potential differences related to sex, the effects of a particular IPP, age-group, and the number of overall training sessions. Age-group was analyzed by comparing studies, which investigated participants being 14 years or younger, with studies investigating participants being older than 14 years of age. This cut-off was chosen based on the available studies in order to arrive at two distinct age-groups. Studies with overlapping age ranges were not considered in this analyses. For the analysis regarding the overall training sessions, we divided the study sample according to the median split technique. All meta-analytical subgroup and exploratory analyses were only conducted, if at least two data points were available (Valentine et al., 2010). In all exploratory analyses, we carefully rated effects being different as indicated by a qualitative analysis of the change magnitude (i.e., effects being small, moderate, or large).

## Results

### Trial flow

**Figure 1:**
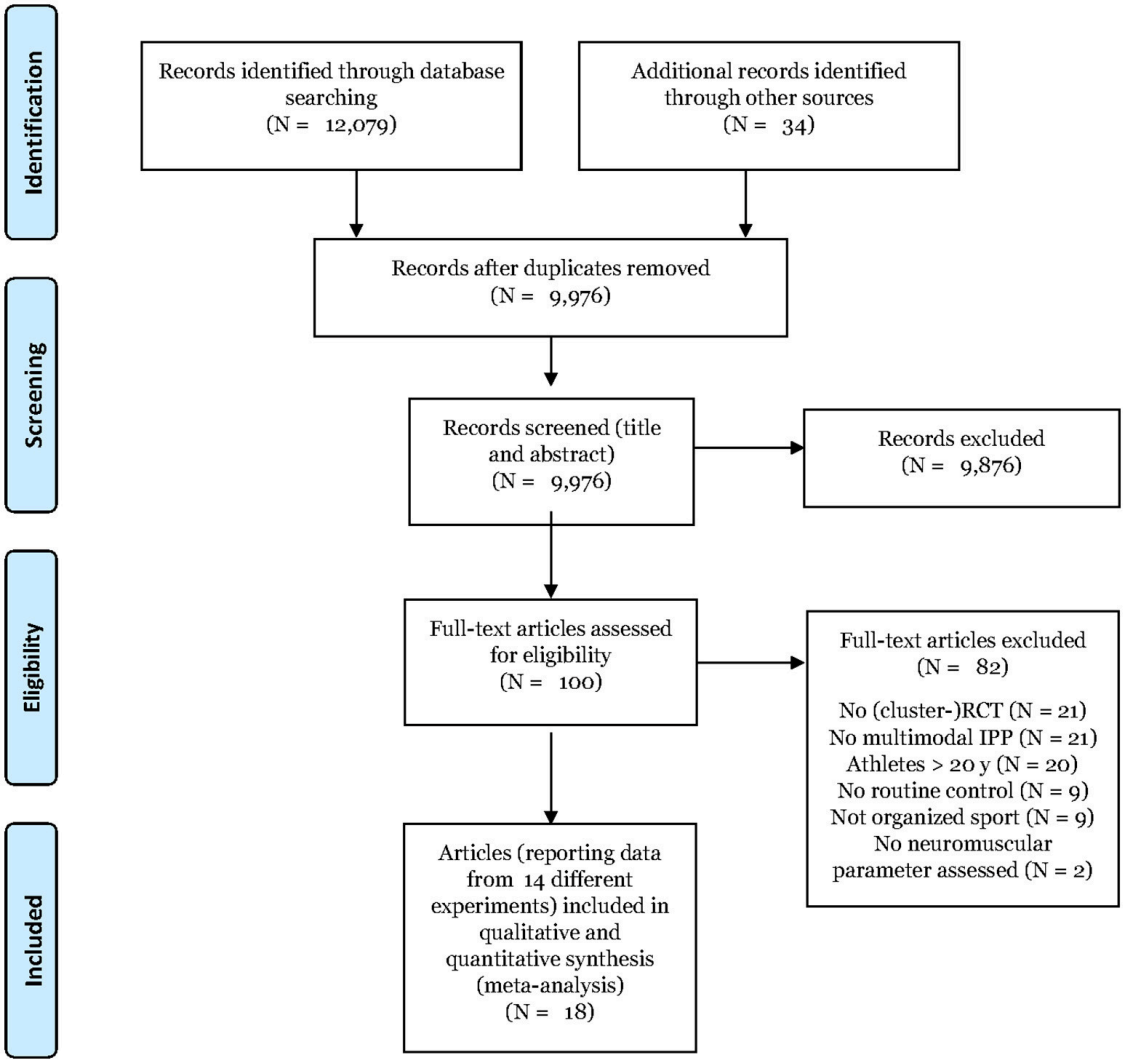
PRISMA flow diagram.

In total, 12,113 potentially relevant articles were initially found (Figure 1). After removing duplicates, 9,976 article titles and abstracts were carefully screened for relevance. The full-texts of the remaining 100 potentially relevant articles were thoroughly studied and 82 papers were excluded as not meeting the inclusion or fulfilling the exclusion criteria.

### Study characteristics

In total, data from 14 different RCTs (comprising 704 participants) published in 18 different scientific articles were finally included in the quantitative meta-analysis (Table 1). All studies were published in 2008 or later. Seventy-one percent of all comparisons analyzed soccer players with basketball, field hockey, futsal, Gaelic football, and hurling being the remaining sports. The average age of the study populations ranged from 10 years up to 19 years and the level of play from low amateur to professional. Intervention duration ranged from 4 weeks to 4.5 months with a total of 12 to maximal 57 training sessions.

**Table 1:** Characteristics of included studies.

**Study; publication date; country**	**Design**	**Participants (analyzed); sport; age; level of play**	**Parameters (included in quantitative analyses)**	**Intervention**
Ayala et al., 2017 Spain	RCT	*N* = 40 male soccer players; 16.8 (SD 0.7) y; first national juvenile league	Y-balance test; 10 and 20 m sprint; Drop jump; Illinois agility test	“11+” vs. Harmoknee vs. control; 4 weeks, 3 × per week (max. 12 sessions)
Daneshjoo et al., 2012a,b, 2013 Iran	RCT	*N* = 36 male soccer players; 17 to 20 y; professional level (daily training)	Isokinetic hamstring and quadriceps strength (ratios); Single leg stance; Star excursion balance test; 20 m sprint; Squat jump; Illinois agility test; Wall-volley test; Slalom dribble	“11+” vs. Harmoknee vs. control; 8 weeks, 3 × per week (max. 24 sessions)
DiStefano et al., 2010 USA	Cluster-RCT	*N* = 65 soccer players (*N* = 39 boys; *N* = 28 girls); 10 (SD 1) y; local soccer association	Time-to-stabilization; Countermovement jump	Pediatric vs. traditional IPP (PEP) vs. control; 9 weeks, 3 × per week (max. 27 sessions)
Heleno et al., 2016 Brazil	RCT	*N* = 22 male soccer players; 14 to 16 y; State and national competitions (5 training sessions per week)	Y-balance test; Single leg stance	Sensorimotor + plyometric IPP vs. control; 5 weeks, 3 × per week (max. 15 sessions)
Kilding et al., 2008 New Zealand	RCT	*N* = 24 male soccer players; 10.4 (SD 1.4) y; Local soccer club	Countermovement jump; 3-step jump; 20 m sprint; Illinois agility test	“The 11” vs. control; 6 weeks, 5 × per week (once per week supervised; max. 30 sessions)
Lim et al., 2009 Korea	RCT	*N* = 22 female basketball players; 15 to 17 y; Highschool basketball	Countermovement jump	Modified PEP vs. control; 8 weeks, frequency n.a.
Lindblom et al., 2012 Sweden	Cluster- RCT	*N* = 41 female soccer players; 12 to 16 y; Local soccer clubs (2 training sessions per week)	Star excursion balance test; Countermovement jump; 3-step jump; 10 and 20 m sprint; Modified Illinois agility test	Knäkontroll vs. control; 11 weeks, 2 × per week (max. 22 sessions)
O'Malley et al., 2017 Ireland	Cluster- RCT	*N* = 56 male Gaelic football and hurling players; 18.1 to 18.8 y; First year collegiate level	Y-balance test	“GAA 15” vs. control; 8 weeks, 2 × per week (max. 16 sessions)
Reis et al., 2013 Portugal	RCT	*N* = 36 male futsal players; 17.3 (SD 0.7) y; 5.8 h futsal activity per week	Isokinetic hamstring and quadriceps strength (ratios); 5 and 30 m sprint; T-test; Countermovement jump; Single leg stance; Slalom dribble	“11+” vs. control; 12 weeks, 2 × per week (max. 24 sessions)
Rössler et al., 2016 Switzerland	Cluster- RCT	*N* = 122 male soccer players; 7 to 13 y; 2 training sessions per week	Single leg stance; Y-balance test; Drop and countermovement jump; Standing long jump; 20 m sprint; Agility parcours; Slalom dribble; Wall-volley test	“11+ Kids” vs. control (sham treatment); 10 weeks, 2 × pre week (max. 20 sessions)
Steffen et al., 2008 Norway	RCT	*N* = 31 female soccer players; 17.1 (SD 0.8) y; Elite sport high schools, competitive level (13.3 h soccer activities per week)	Isokinetic and isometric hamstring and quadriceps strength (ratios); Drop and countermovement jump; 40 m sprint; Slalom dribble	“The 11” vs. control; 10 weeks, 3 × per week (max. 30 sessions)
Steffen et al., 2013 Canada	Cluster- RCT	*N* = 148 female soccer players; 13 to 18 y; Minor and youth soccer associations, 3 highest levels	Single leg stance; 3-step jump; Y-balance test	Regular “11+” vs. control; 4.5 months; 2–3 × per week (max. 57 sessions)
Vescovi and VanHeest, 2010 USA	Cluster- RCT	*N* = 31 female soccer players; 13 to 18 y; Local soccer community (3 training sessions per week)	9.1, 18.3, 27.4 and 36.6 m sprint; Countermovement jump; Modified Illinois agility test	PEP vs. control; 12 weeks, 3 × per week (max. 36 sessions)
Zech et al., 2014 Germany	RCT	*N* = 30 male field hockey players; 14.9 (SD 3) y; Highest regional youth division (2 training sessions per week)	Y-balance test; Time-to-stabilization	Neuromuscular IPP vs. control; 10 weeks, 2 × per week (max. 20 sessions)

*SD, standard deviation; RCT, randomized controlled trial; IPP, injury prevention program; PEP, prevent injuries enhance performance*.

Four studies were classified as high-level (Steffen et al., 2008; Daneshjoo et al., 2012a,b, 2013; Heleno et al., 2016; Ayala et al., 2017), eight as low-level (Kilding et al., 2008; DiStefano et al., 2010; Vescovi and VanHeest, 2010; Lindblom et al., 2012; Reis et al., 2013; Steffen et al., 2013; Zech et al., 2014; Rössler et al., 2016) and in two studies (Lim et al., 2009; O'Malley et al., 2017) the performance level could not be definitely estimated and, thus, these studies were not included in this particular sub-analysis.

Eight studies reported data on boys only (Kilding et al., 2008; Daneshjoo et al., 2012a,b, 2013; Reis et al., 2013; Zech et al., 2014; Heleno et al., 2016; Rössler et al., 2016; Ayala et al., 2017; O'Malley et al., 2017) and five studies on girls only (Steffen et al., 2008, 2013; Lim et al., 2009; Vescovi and VanHeest, 2010; Lindblom et al., 2012).

The following IPP were analyzed in at least two studies (Valentine et al., 2010): “11+” (Daneshjoo et al., 2012a,b, 2013; Reis et al., 2013; Steffen et al., 2013; Ayala et al., 2017), “The 11” (Kilding et al., 2008; Steffen et al., 2008), “HarmoKnee” (Daneshjoo et al., 2012a,b, 2013; Ayala et al., 2017) or the “Prevent Injury Enhance Performance” program (PEP or a modified version; Lim et al., 2009; DiStefano et al., 2010; Vescovi and VanHeest, 2010). Further programs used either a combination of exercises extracted from these established programs (Zech et al., 2014; O'Malley et al., 2017), adapted versions for younger children (DiStefano et al., 2010; Rössler et al., 2014) or applied sensorimotor training combined with plyometrics (Heleno et al., 2016).

Three studies analyzed participants being 14 years or younger (Kilding et al., 2008; DiStefano et al., 2010; Rössler et al., 2016) and in six studies athletes were 15 years or older (Steffen et al., 2008; Lim et al., 2009; Daneshjoo et al., 2012a,b, 2013; Reis et al., 2013; Ayala et al., 2017; O'Malley et al., 2017).

Seven studies had a large (>23 sessions; Kilding et al., 2008; Steffen et al., 2008, 2013; DiStefano et al., 2010; Vescovi and VanHeest, 2010; Daneshjoo et al., 2012a,b, 2013; Reis et al., 2013) and the remaining studies a low (<23 sessions; Lindblom et al., 2012; Zech et al., 2014; Heleno et al., 2016; Rössler et al., 2016; Ayala et al., 2017; O'Malley et al., 2017) number of training sessions during the study period.

### Risk of bias

The funnel plot evaluation (Appendix 3 in Supplementary Material) showed no obvious risk of bias in balance/stability, leg power, and strength measures. In sprint abilities and sport-specific tests a slight overrepresentation of small studies with large effects seems apparent.

The results of the study quality assessment are presented in Appendix 4 (Supplementary Material). Seven studies obtained the PEDro score 7 (Steffen et al., 2008; DiStefano et al., 2010; Zech et al., 2014; Heleno et al., 2016; Rössler et al., 2016; Ayala et al., 2017; O'Malley et al., 2017), six studies a score of 6 (Kilding et al., 2008; Lim et al., 2009; Vescovi and VanHeest, 2010; Daneshjoo et al., 2012a,b, 2013; Lindblom et al., 2012; Reis et al., 2013) and one study was rated as PEDro 5 (Steffen et al., 2013).

When comparing studies, which were rated as PEDro 7, with all other studies, we observed no relevant differences between “strong” and “weak” studies in balance/stability, leg power, and sprint abilities, whereas effects were larger in the “weak” studies for isokinetic strength and sport-specific tests (Appendix 5 in Supplementary Material). However, the latter analyses were based on merely two “strong” studies (Steffen et al., 2008; Rössler et al., 2016).

### Main analysis—intervention effects

**Figure 2:**
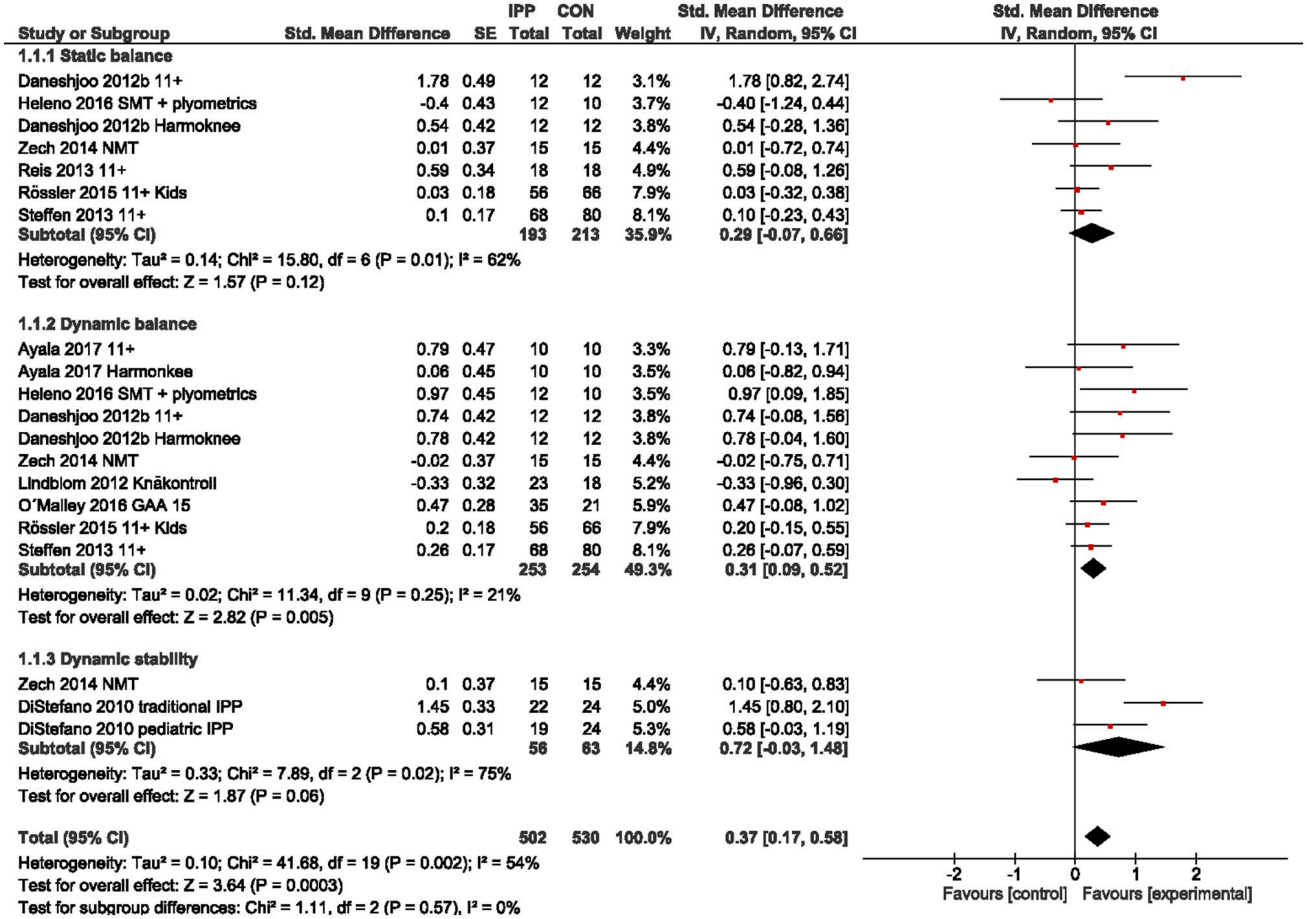
Standardized mean effects of injury prevention programs (IPP) on balance and stability parameters as compared to a control (CON) group. Data are separately presented for static and dynamic balance as well as dynamic stability measures. SE, standard error; IV, inverse variance model; CI, confidence interval; SMT, sensorimotor training; NMT, neuromuscular training.

**Figure 3:**
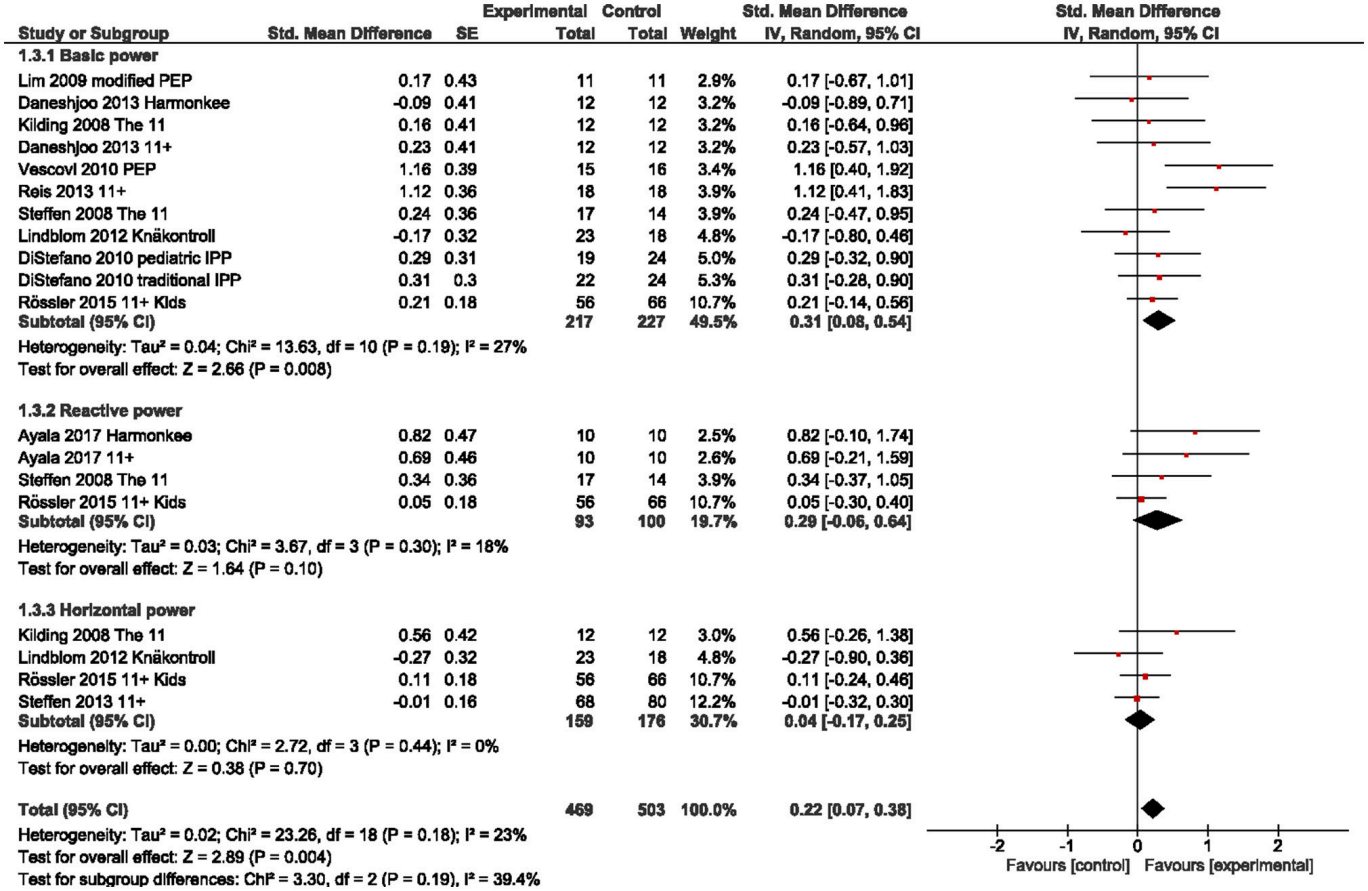
Standardized mean effects of injury prevention programs (IPP) on leg power parameters as compared to a control (CON) group. Data are separately presented for basic, reactive and horizontal power measures. SE, standard error; IV, inverse variance model; CI, confidence interval; PEP, Prevent Injury Enhance Performance.

**Figure 4:**
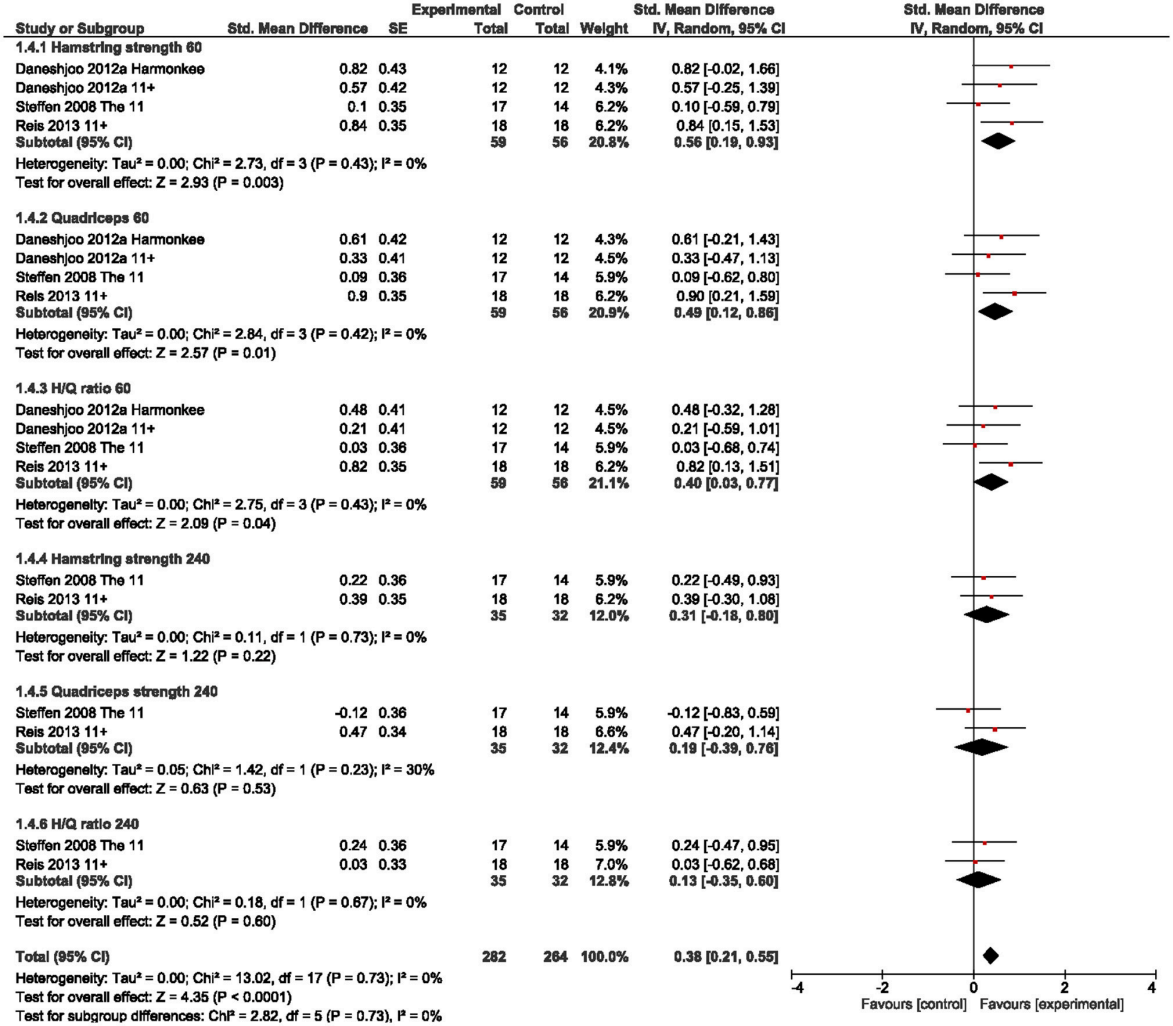
Standardized mean effects of injury prevention programs (IPP) on leg isokinetic strength as compared to a control (CON) group. Data are separately presented for hamstring (H) and quadriceps (Q) strength as well as H/Q ratios at movement velocities of 60 and 240°/s. SE, standard error; IV, inverse variance model; CI, confidence interval.

We observed a small overall effect in favor of IPP for balance/stability outcomes including static and dynamic balance measures [g = 0.37 (95%CI 0.17, 0.58); Figure 2]. For dynamic stability measures we found a moderate effect (g = 0.72), but CI overlap zero. Similarly, a small overall effect was present for leg power outcomes [g = 0.22 (95%CI 0.07, 0.38); Figure 3], particularly for basic (g = 0.31) and reactive power (g = 0.29) parameters, but not for horizontal power (g = 0.04). Further, we found small to moderate effects for isokinetic hamstrings (g = 0.56) and quadriceps strength (g = 0.49) as well as for hamstrings-to-quadriceps ratio (g = 0.40) at low movement speed (60° per second), but not at fast movement speed (240° per second; g = 0.13 to 0.31; Figure 4).

**Figure 5:**
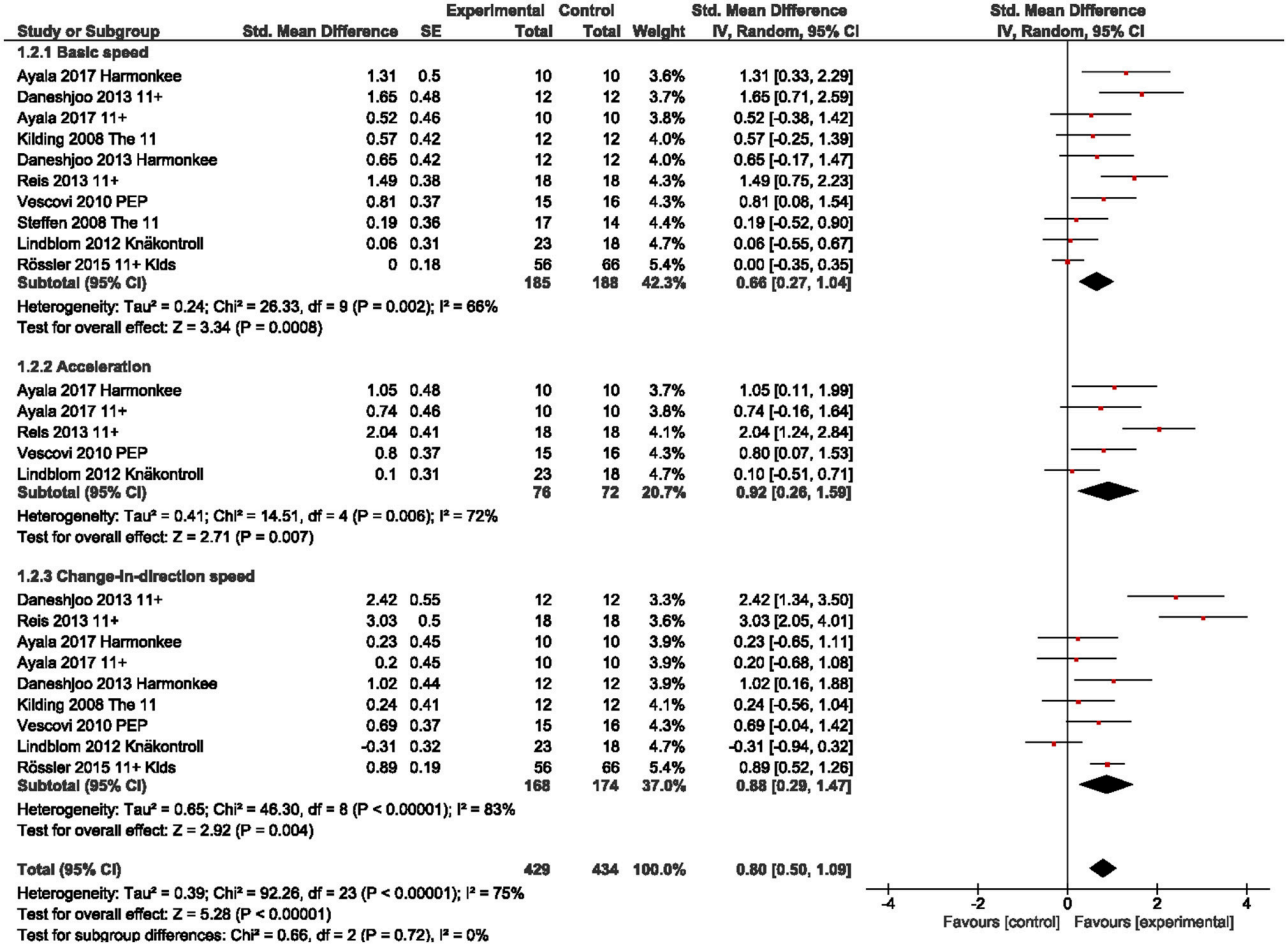
Standardized mean effects of injury prevention programs (IPP) on sprint abilities as compared to a control (CON) group. Data are separately presented for basic speed, acceleration and change-in-direction speed. SE, standard error; IV, inverse variance model; CI, confidence interval; PEP, Prevent Injury Enhance Performance.

**Figure 6:**
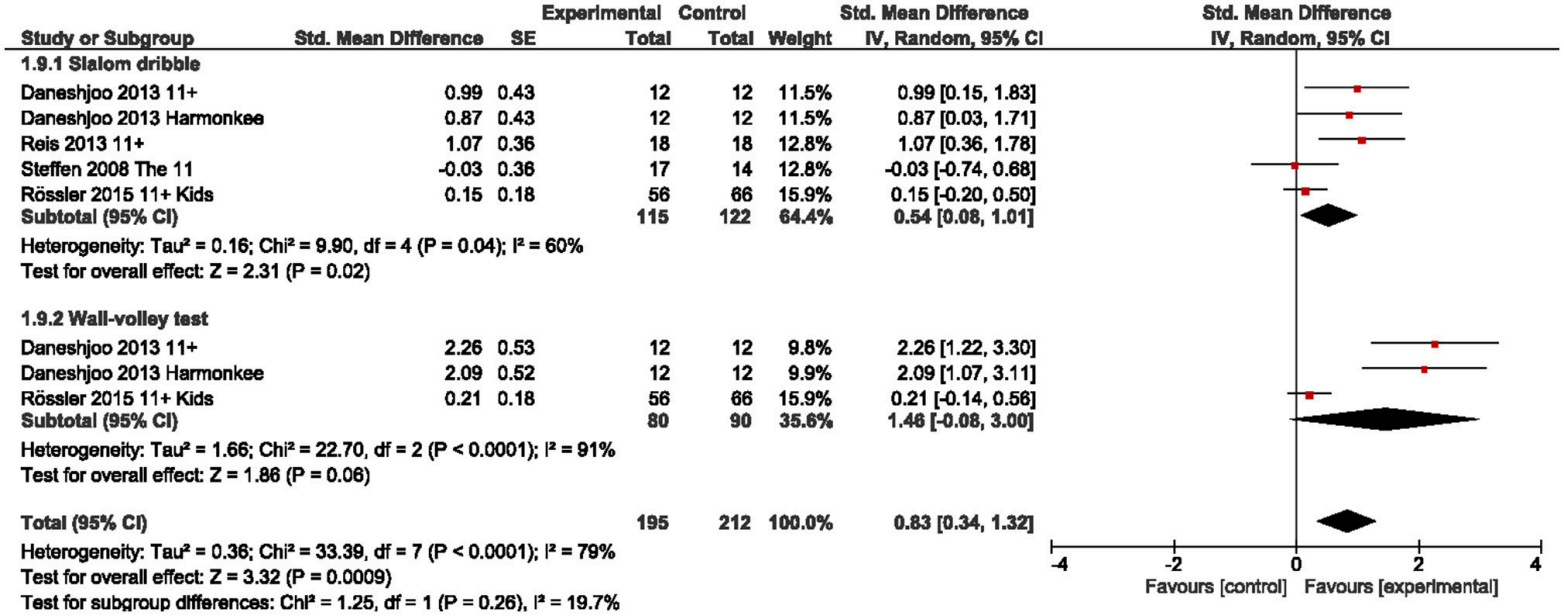
Standardized mean effects of injury prevention programs (IPP) on sport-specific skills as compared to a control (CON) group. Data are separately presented for slalom dribbling and the wall-volley test. SE, standard error; IV, inverse variance model; CI, confidence interval.

We observed a large overall effect for sprint abilities [g = 0.80 (95%CI 0.50, 1.09); Figure 5], which was particularly present in acceleration (g = 0.92) and change-in-direction speed (g = 0.88). Basic speed abilities were moderately improved (g = 0.66). With regard to sport-specific skills (here: soccer-specific) we found a moderate effect for slalom dribbling (g = 0.54) and a large effect for the wall-volley test (g = 1.46) with CI including the zero (Figure 6).

### Subgroup and exploratory analyses

Table 2 displays the effects for the different levels of play. We found consistently larger effects in the high-level group (g = 0.34–1.18) compared to the low level group (g = 0.22–0.75), with large effects in sprint abilities and sport-specific tests and moderate effects in balance/stability measures. The low-level group showed small to moderate effects in all categories. However, CI largely overlapped.

**Table 2:** Training adaptations (pooled standardized mean differences with 95% confidence intervals; qualitative assessment of effect magnitude) for high- vs. low-level players.

	**High-level players**	**Low-level players**
Balance/stability	**0.64** (95% CI 0.21,1.07); **moderate**	0.25 (95% CI 0.02,0.47); small
Leg power	**0.34** (95% CI 0.01,0.66); **small**	0.22 (95% CI 0.02,0.42); small
Sprint abilities	**0.86** (95% CI 0.47,1.24); **large**	0.75 (95% CI 0.33,1.17); moderate
Sport-specific tests	**1.18** (95% CI 0.34,2.02); **large**	0.37 (95% CI −0.05,0.79); small

*Bold values indicate larger effects for the corresponding measure*.

Boys showed small to moderate effects for balance/stability, leg strength and power, whereas girls showed negligible to small effects (Table 3). Particularly for sprint abilities, we found large effects in boys. With regard to the different IPP, we found small to moderate effects for “11+” and “HarmoKnee” in balance/stability as well as leg power and strength parameters and large effects for sprint abilities and sport-specific skills for these IPP (Table 4). PEP showed moderate effects for leg power and sprint abilities. The effects for “The 11” were negligible to small. While we observed similar moderate effects in balance/stability measures in both the younger and older age-group, the older athletes showed a large effect in sprint abilities (Table 5). The studies with <23 training sessions showed in all categories negligible to small effects, whereas those studies with more than 23 sessions showed a small effect in leg power, a moderate effect in balance/stability and large effects in sprint abilities and sport-specific skills (Table 6).

**Table 3:** Training adaptations (pooled standardized mean differences with 95% confidence intervals; qualitative assessment of effect magnitude) between sexes.

	**Girls**	**Boys**
Balance/stability	**0.38** (95% CI −0.07,0.84); **small**	0.37 (95% CI 0.14,0.60); small
Leg power	0.15 (95% CI −0.16,0.46); negligible	**0.27** (95% CI 0.07,0.48); **small**
Isokinetic leg strength	0.09 (95% CI −0.19,0.38); negligible	**0.54** (95% CI 0.32,0.75); **moderate**
Sprint abilities	0.30 (95% CI–0.02,0.62); small	**1.02** (95% CI 0.64,1.40); **large**
Sport-specific tests	–	0.97 (95% CI 0.42,1.51); large

*Bold values indicate larger effects for the corresponding measure*.

**Table 4:** Training adaptations (pooled standardized mean differences with 95% confidence intervals; qualitative assessment of effect magnitude) for the different injury prevention programs.

	**“11+”**	**“The 11”**	**HarmoKnee**	**PEP**
Balance/stability	**0.55** (95% CI 0.17,0.94); **moderate**	–	0.48 (95% CI −0.01,0.96); small	–
Leg power	0.45 (95% CI −0.12,1.02); small	0.32 (95% CI −0.06,0.69); small	0.34 (95% CI −0.55,1.23); small	**0.54** (95% CI −0.04,1.12); **moderate**
Isokinetic leg strength	0.51 (95% CI 0.27,0.75); moderate	0.09 (95% CI −0.19,0.38); negligible	**0.63** (95% CI 0.16,1.11); **moderate**	–
Sprint abilities	**1.49** (95% CI 0.84,2.14); **large**	0.03 (95% CI −0.13,0.76); negligible	0.82 (95% CI 0.43,1.22); large	0.77 (95% CI 0.35,1.19); moderate
Sport-specific tests	1.37 (95% CI 0.65,2.08); large	–	**1.44** (95% CI 0.25,2.64); **large**	–

*Bold values indicate the largest effect for the corresponding measure. PEP, prevent injury enhance performance*.

**Table 5:** Training adaptations (pooled standardized mean differences with 95% confidence intervals; qualitative assessment of effect magnitude) between young and old players.

	**Players < 15 years**	**Players ≥ 15 years**
Balance/stability	0.51 (95% CI −0.02,1.03); moderate	**0.66** (95% CI 0.37,0.95); **moderate**
Leg power	0.18 (95% CI 0.00,0.35); negligible	**0.32** (95% CI 0.05,0.60); **small**
Sprint abilities	0.43 (95% CI −0.10,0.95); small	**1.16** (95% CI 0.72,1.60); **large**
Sport-specific tests	0.18 (95% CI −0.07,0.43); negligible	**1.15** (95% CI 0.49,1.81); **large**

*Bold values indicate larger effects for the corresponding measure*.

**Table 6:** Training adaptations (pooled standardized mean differences with 95% confidence intervals; qualitative assessment of effect magnitude) relative to the total number of training sessions.

	**<23 Training sessions**	**>23 Training sessions**
Balance/Stability	0.14 (95% CI −0.05,0.33); negligible	**0.67** (95% CI 0.33,1.01); **moderate**
Leg power	0.12 (95% CI −0.07,0.31); negligible	**0.35** (95% CI 0.10,0.59); **small**
Sprint abilities	0.38 (95% CI 0.07,0.69); small	**1.16** (95% CI 0.72,1.59); **large**
Sport-specific tests	0.18 (95% CI −0.07,0.43); negligible	**1.15** (95% CI 0.49,1.81); **large**

*Bold values indicate larger effects for the corresponding measure*.

## Discussion

The main aim of present meta-analysis was to summarize the scientific literature on neuromuscular performance adaptations resulting from multimodal IPP in organized child and adolescent sport and to quantify the effect sizes of adaptations in various neuromuscular performance measures. Furthermore, we performed subgroup analyses regarding potential differences in adaptations between performance levels, sex, age-groups, specific IPP, and number of training sessions.

### Key results

With regard to our main study question we found that multimodal IPP improved several neuromuscular performance measures. We observed small effects for balance/stability measures as well as leg power and a medium effect for isokinetic leg strength at low movement velocities. For sprint abilities and sport-specific tests we found large effects.

Regarding subgroup and exploratory analyses, we obtained slightly larger effects in athletes of a higher performance level. There were differences in adaptations between different IPP, greater adaptations in boys, older players and in studies with higher number of training sessions during the study period.

### Overall interpretation and generalizability

Neuromuscular deficits, for instance regarding balance, stability, leg power, and leg strength, are considered potential intrinsic risk factors for injuries (Meeuwisse, 1994; Bahr and Holme, 2003; Myer et al., 2011; Lehr et al., 2017). These risk factors are modifiable by appropriate neuromuscular training regimens (Alentorn-Geli et al., 2009; Myer et al., 2011; Bizzini and Dvorak, 2015). IPP, which have been shown to reduce injuries, target these risk factors within a multimodal training approach (Mandelbaum et al., 2005; Soligard et al., 2008; Kiani et al., 2010; Walden et al., 2012). Our analysis showed that parameters in relevant neuromuscular domains, such as, balance, postural stability, or leg strength and power, notably benefit from IPP. Effects were, however, small to moderate. It can be speculated, that small effects may be sufficient to relevantly reduce the risk for injury, particularly, as small effects in different domains (e.g., power, strength, balance) may act synergistically (Myer et al., 2005). For instance, an improvement of neuromuscular joint control, e.g., resulting from slightly improved balance and an increased strength of thigh musculature, may reduce forces and moments on muscles and ligaments in situations with high biomechanical loads (e.g., cutting maneuvers) sufficiently in order to avoid traumatic events. Interestingly, time-to-stabilization after single leg landing as an indicator of the ability to stabilize the body in a dynamic situation showed a large effect. As time-to-stabilization was assessed in only two studies (DiStefano et al., 2010; Zech et al., 2014), the confidence interval slightly overlapped the zero and the reliable assessment is methodologically and technically challenging, this result should be cautiously interpreted. However, it might be regarded as an interesting parameter for future injury prevention studies as exercises including one leg standing and landing situations are a main part of IPP. Similarly, we also found large improvements in agility tests. Such tests aim at rapid de- and accelerations while changing movement direction, i.e., the ability to stabilize the joints under large biomechanical loads. Such an ability may allow for an efficient transfer of forces and moments and, consequently, has high relevance for performance and likely also for the prevention of injuries.

In addition, we also observed large improvements in straight sprinting speed as well as in the ability to accelerate rapidly. Whereas, it is questionable whether straight sprinting is relevant from an injury prevention perspective, it is generally considered one of the most important physical abilities in team sports from a performance perspective. For instance, Faude et al. (2012) showed that straight sprinting is the most important powerful action preceding goal situations in professional soccer. Particularly, the “11+” program revealed large effects on sprint performance. This may be due to exercises such as, the Nordic hamstrings, plyometrics, or the bounding jumps. Evidence suggests that these exercises and, particularly, combinations of it can effectively improve sprinting abilities (Rumpf et al., 2016). As many coaches in team sports accentuate the development of sprinting speed, our findings may contribute to convincing coaches to implement IPP in their training routine.

Improvements in sport-specific tests like slalom dribbling or the wall-volley test may have a similar effect on coaches' willingness to implement IPP. The underlying reasons why athletes enhance their sport-specific skills through IPP is unclear. One might speculate that improved neuromuscular control during sport-specific skills may enable athletes to better and faster process the ball and, thus, have more attentional capacity to control movements. In this regard it is interesting to note that inappropriately developed sport-specific skills are also considered a potential intrinsic risk factor (Meeuwisse, 1994; Bahr and Holme, 2003). Therefore, the observed large improvements in sport-specific skills may contribute to a decreased injury risk.

IPP are frequently designed as warm-up programs lasting about 15–20 min. Bizzini et al. (2013) showed, for instance, that the “11+” fulfills the requirements of a warm-up program in soccer players. Furthermore, there is a large body of evidence that the “11+” program can reduce injury rate considerably (Barengo et al., 2014; Bizzini and Dvorak, 2015; Thorborg et al., 2017). Taken this evidence together, appropriately designed IPP lasting only 15–20 min per session can serve as appropriate warm-up programs. Thereby, these programs are able to improve potential risk factors for injury as well as performance indicators and, simultaneously, to reduce injury rate. This might be a strong point toward a broad implementation of such programs in sports practice.

An interesting finding was that high-level players showed slightly larger effects compared to low-level players. Intuitively, one would assume that the adaptive potential in low-level players is greater and, consequently, adaptations in these players should have been larger. In high-level players a ceiling effect in training adaptability seems reasonable as the stimuli provided by IPP may not be appropriate to induce further improvements. Our results are contradictory to these assumptions. We cannot definitely conclude on the underlying reasons for this finding. It might be speculated that in the high-level teams the coaching staff is better qualified and, thus, the training stimulus was applied in a more appropriate or suitable manner. In contrast, coaches on lower levels of play are frequently not appropriately qualified and, hence, it might be more difficult for them to instruct a correct execution of exercises for the sake of an adequate training stimulus. However, the results should be carefully interpreted as CI were wide and overlapping. Therefore, future research seems necessary.

We found also larger effects in older players as compared to their younger counterparts. This result might be related to the differences in adaptations with different playing levels as the high-level players were older (14–20 years) than the low-level players (7–18 years). Therefore, we cannot definitely distinguish between both factors. Age has been consistently shown to be an important non-modifiable risk factor for injuries (Emery, 2003). Thus, the observed adaptations in the older players might be particularly relevant from an injury prevention perspective.

We obtained larger effects in boys than in girls, particularly, in isokinetic leg strength and sprint abilities. The performance effects in girls were negligible to small. A recent meta-analysis (Rössler et al., 2014) has shown that IPP are efficacious in organized youth sports in both sexes. Based on our results we cannot conclude on possible mechanisms for this finding. Faude et al. (2013) reported that joint-ligament injuries (sprains), particularly knee sprains, are more frequent in girls compared to boys in youth soccer. Thus, injury prevention studies in female youth sports often focus on knee injuries, whereas this is not the case in studies on boys (Rössler et al., 2014). One might speculate that anatomical and biomechanical risk factors (leg alignment, knee valgus moments, knee internal rotation, cutting task biomechanics, etc.) contribute more to the risk for knee injuries and, consequently, are more important regarding injury prevention in females. There is evidence that such biomechanical risk factors are also modifiable by IPP (Pappas et al., 2015). Future research regarding sex-specific training adaptations is warranted.

When comparing the different IPP it is obvious that the performance effects were comparable between the “11+,” the “HarmoKnee” and the PEP program. The effects of the “The 11,” the predecessor of the “11+,” were considerably smaller. This finding is in line with meta-analytical data showing that the application of “11+” resulted in large reductions in injury incidence, whereas the preventative efficacy of “The 11” could not be substantiated (Al Attar et al., 2016; Thorborg et al., 2017).

Regarding the total number of training sessions our results clearly demonstrate that the effect is larger as with a greater total training volume. Thus, it is advisable to conduct IPP over a longer period of time in order to increase efficacy. This finding strengthens the results of Sugimoto et al. (2014) showing that there is a relationship between dosage and ACL injury reduction in female athletes. Similarly, Lesinski et al. (2015) showed that strength training adaptations in youth athletes are larger when training duration exceeded 23 weeks emphasizing the relevance of longer training durations. There is also evidence that training compliance had a relevant effect on program efficacy (Sugimoto et al., 2012). Similarly, Steffen et al. (2013) showed that a high compliance to the “11+” program resulted in improvements in functional balance and a reduced injury risk. Currently, it is recommended to perform the program at least 1.5 times per week in order to optimize training effectiveness (Barengo et al., 2014). These recommendations were exceeded by all studies included in our analyses. Information on the compliance in the included studies was limited. Thus, we cannot conclude on the influence of compliance on our results. It has to be mentioned that the evidence on the appropriate training frequency is currently limited.

### Methodological considerations

Within the present meta-analysis, we focused on (cluster-) RCTs published in peer-reviewed scientific journals. All studies were of sufficient to high methodological quality (PEDro score ≥ 5). Thus, the present results are based on high-quality research. We did not include non-randomized studies or gray literature, thus, accepting the potential for a publication bias. However, a risk of bias analysis did only show a slight risk for the sprint and sport-specific test categories. We analyzed studies, which used evidence-based IPP or blends of such programs. Studies applying other neuromuscular or strength and conditioning programs focusing on performance improvements and not on injury prevention were not analyzed, although such programs also have the potential to reduce injury risk in youth sport (Myer et al., 2011; Granacher et al., 2016). Most studies in our analysis were conducted in soccer. Evidence in other sports and the transferability of our results is, therefore, currently limited. Finally, it has to be mentioned that the statistical power of our analyses was low for some subgroup and exploratory analyses. As CI were large and overlapping, these results should be carefully interpreted. Also, the separation of age-groups in being older and younger than 14 years of age was due to practicality. It would have been better to apply a measure of maturational status, but such information was not available.

## Conclusions, practical implications and perspectives

Multimodal IPP, which have been shown to reduce the risk of injury, improve several neuromuscular performance parameters, which have been associated with injury risk. Although effects were partly small, this may give an explanation for the preventative efficacy of these programs. The general improvements in neuromuscular performance may also support the wide-spread implementation and dissemination of IPP as performance improvements are a strong argument for coaches to use particular training programs. The implementation of IPP as a routine 15–20-min warm-up can effectively prepare for the following training session, can improve performance and reduce the risk of injury. The optimal IPP may fulfill the following requirements: (i) It should be effective with regard to injury prevention and performance enhancement; (ii) it should be efficient with regard to time and resources needed to apply the program; (iii) it should be feasible and practical; (iv) it should be specific, i.e., targeted with regard to the specific sport, age, sex, and performance level.

Future research is needed to analyze which parts of multimodal programs are most effective. This may allow for optimizing existing programs or making them more efficacious and time efficient. Further, it may allow for adapting programs to other sports and settings. Furthermore, it seems warranted to adapt programs particularly for girls in order to improve performance parameters also in female youth athletes. Studies in younger children are underrepresented. Injury incidence in the youngest children is lower as compared to older athletes and injury characteristics partly differ (Faude et al., 2013). Only two studies used a program, which was specifically adapted for the youngest athletes. This is of particular interest as Myer et al. (2013) suggested to introduce prevention programs before the onset of neuromuscular deficits. Early preventive measures might be important as children make up a large part of the active population, as it may be of particular importance to prevent or at least delay the first injury and as it may support the sensitization of young athletes with injuries and appropriate preventive means.

## Author contributions

OF had the idea, designed and conducted this meta-analysis. RoR, EP, RaR, LZ, and LD assisted in the design of this study. OF, LD, and RoR developed the search strategy. LD and OF conducted the literature search and the study quality assessment. RoR and OF extracted study information and outcome data. OF performed the statistical analyses and wrote the first paper draft. All authors revised the manuscript for important intellectual content and approved the final version of the article.

### Conflict of interest statement

The authors declare that the research was conducted in the absence of any commercial or financial relationships that could be construed as a potential conflict of interest.
