# Supplementary material for: Neuromuscular Adaptations to Multimodal Injury Prevention Programs in Youth Sports: A Systematic Review with Meta-Analysis of Randomized Controlled Trials
Source: Front Physiol. 2017 Oct 12;8:791. doi: 10.3389/fphys.2017.00791 (PMC5643472; doi:10.3389/fphys.2017.00791)
Supplement: Supplementary file 1 [file DataSheet1.docx]

**Appendix 1.** Search strategy until May 8^th^ 2017.

**„Neuromuscular adaptations to multimodal injury prevention programs in youth sports: A systematic review with meta-analysis of randomized controlled trials“**

|  | **searches** | | |  |  |
| --- | --- | --- | --- | --- | --- |
| **database** | **#1** | **#2** | **#3** | | **Screened titles** (after duplicates removed) |
| CINAHL* | 197,379 | 31,756 | 1,777 | | 804 |
| EMBASE** | 46,394 | 12,442 | 955 | | 499 |
| ISI Web of Knowledge*** | 621,697 | 24,610 | 348 | | 185 |
| PubMed**** | 1,027,605 | 166,703 | 5,962 | | 5,954 |
| Scopus***** | 577,655 | 119,140 | 3,037 | | 2,498 |
| sum | 2,470,730 | 354,651 | 12,079 | | 9,942 |
|  |  |  |  | |  |

| Search terms #1 | youth OR junior OR child* OR adolescen* OR high school OR college |
| --- | --- |
| Search terms #2 | #1 AND injur* OR prevent* OR neuromuscular OR multimod* OR exercise OR train* OR plyometric* |
| Search terms #3 | #2 AND strength OR speed OR sprint OR agility OR balance OR motor performance |

* Search in title and abstract; English and German; Peer-reviewed articles; Human

** Search in title and abstract; limited to randomized controlled trials

*** Search in title; English and German; Articles

**** Search in title and abstract; English and German; Journal articles; Human

***** Search in title, abstract and key words; English and German; Journal articles

**Appendix 2.** Categorization of neuromuscular performance parameters.

| Category | Sub-category | Test |
| --- | --- | --- |
| Balance/stability | Static balance | Single leg stance |
|  | Dynamic balance | Y-balance test  Star excursion balance test |
|  | Dynamic stability | Time-to-stabilization |
| Leg power | Reactive vertical power | Drop jump |
|  | Basic vertical power | Countermovement jump  Squat jump |
|  | Horizontal power | Standing long jump  3-step jump |
| Isokinetic leg strength | Isokinetic thigh muscle strength at low movement velocity (60°/s) | Hamstring (H) strength  Quadriceps (Q) strength  H/Q ratio |
|  | Isokinetic thigh muscle strength at fast movement velocity (240°/s) | Hamstring (H) strength  Quadriceps (Q) strength  H/Q ratio |
| Sprint abilities | Acceleration | 5 m straight sprint  9.1 m straight sprint  10 m straight sprint |
|  | Basic speed | 18.3 to 36.6 m straight sprint  20 m straight sprint  30 m straight sprint  40 m straight sprint |
|  | Change-in-direction speed | Agility parcours  T test  (modified) Illinois agility test |
| Sport-specific tests | Passing | Wall-volley test |
|  | Dribbling | Slalom dribble |

**Appendix 3.** Funnel plots for all main analyses.


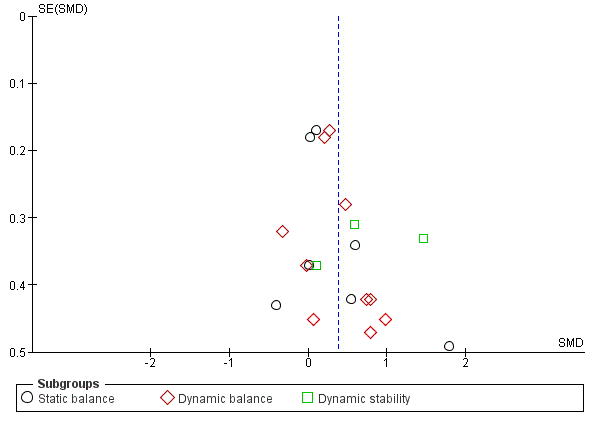

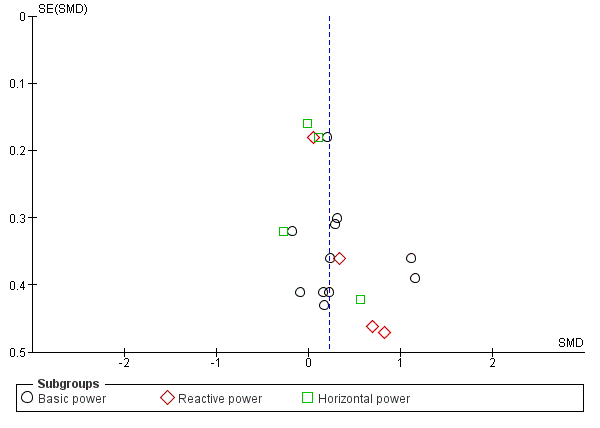

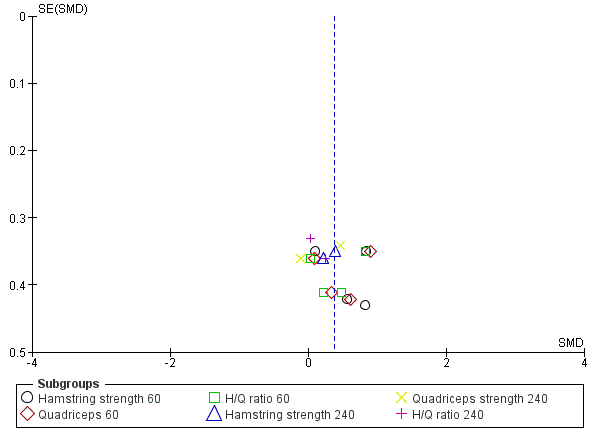

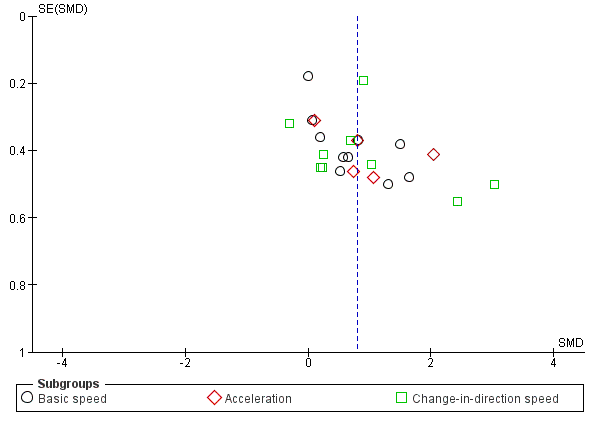

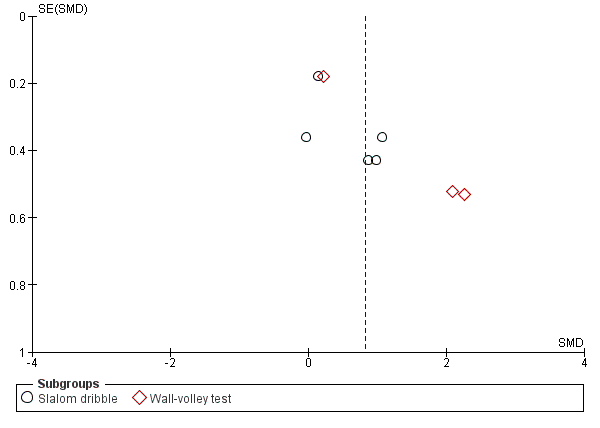


**Appendix 4.** PEDro scale assessment.

| **Author** | **eligibility specified** | **subjects randomly allocated** | **concealed allocation** | **similar baseline values** | **blinding of subjects** | **blinding of therapist** | **blinding of assessor** | **dropout <15%** | **received treatment as allocated** | **statistical between-group comparison** | **point measures and variability provided** | **sum  (2 to 11)** |
| --- | --- | --- | --- | --- | --- | --- | --- | --- | --- | --- | --- | --- |
| **Ayala et al. 2016** | √ | √ | √ | √ | - | - | √ | - | √ | √ | √ | **7** |
| **Daneshjoo et al. 2012/2013** | √ | √ | - | - | - | - | √ | √ | √ | √ | √ | **6** |
| **DiStefano et al. 2010** | √ | √ | √ | √ | - | - | - | √ | √ | √ | √ | **7** |
| **Heleno et al. 2016** | √ | √ | √ | √ | √ | - | - | √ | √ | - | √ | **7** |
| **Kilding et al. 2008** | - | √ | - | √ | - | - | - | √ | √ | √ | √ | **6** |
| **Lim et al 2009** | - | √ | - | √ | - | - | - | √ | √ | √ | √ | **6** |
| **Lindblom et al. (2012)** | √ | √ | √ | √ | - | - | - | - | √ | √ | √ | **6** |
| **O’Malley et al. (2016)** | √ | √ | √ | √ | - | - | √ | - | √ | √ | √ | **7** |
| **Reis et al. (2013)** | - | √ | - | √ | - | - | - | √ | √ | √ | √ | **6** |
| **Rössler et al. (2015)** | √ | √ | √ | √ | √ | - | - | - | √ | √ | √ | **7** |
| **Steffen et al. (2008)** | √ | √ | √ | √ | - | - | - | √ | √ | √ | √ | **7** |
| **Steffen et al. (2013)** | √ | √ | √ | - | - | - | - | - | √ | √ | √ | **5** |
| **Vescovi et al. (2010)** | √ | √ | √ | √ | - | - | - | - | √ | √ | √ | **6** |
| **Zech et al. (2013)** | √ | √ | √ | √ | - | - | - | √ | √ | √ | √ | **7** |

**Appendix 5.** Differences in training adaptations (pooled standardized mean differences with 95% confidence intervals) in “weak” (PEDro score 5 or 6) and “strong” (PEDro score 7) studies.

|  | **PEDro score 5/6** | **PEDro score 7** |
| --- | --- | --- |
| **Balance/stability** | 0.44 (95% CI 0.10,0.78) | 0.34 (95% CI 0.07,0.61) |
| **Leg power** | 0.25 (95% CI -0.06,0.56) | 0.21 (95% CI 0.04,0.38) |
| **Isokinetic leg strength** | 0.54 (95% CI 0.32,0.75) | 0.09 (95% CI -0.19,0.38) |
| **Sprint abilities** | 0.97 (95% CI 0.52,1.41) | 0.52 (95% CI 0.18,0.86) |
| **Sport-specific tests** | 1.38 (95% CI 0.84,1.92) | 0.16 (95% CI -0.08,0.39) |
